# Rapid Reactivation of Cyanobacterial Photosynthesis and Migration upon Rehydration of Desiccated Marine Microbial Mats

**DOI:** 10.3389/fmicb.2015.01472

**Published:** 2015-12-24

**Authors:** Arjun Chennu, Alistair Grinham, Lubos Polerecky, Dirk de Beer, Mohammad A. A. Al-Najjar

**Affiliations:** ^1^Max Planck Institute for Marine MicrobiologyBremen, Germany; ^2^School of Civil Engineering, The University of Queensland, St. LuciaQLD, Australia; ^3^Department of Earth Sciences, Utrecht UniversityUtrecht, Netherlands; ^4^Red Sea Research Center, King Abdullah University of Science and TechnologyJeddah, Saudi Arabia

**Keywords:** cyanobacteria, desiccation tolerance, extreme environment, rehydration, photosynthesis, reactivation

## Abstract

Desiccated cyanobacterial mats are the dominant biological feature in the Earth’s arid zones. While the response of desiccated cyanobacteria to rehydration is well-documented for terrestrial systems, information about the response in marine systems is lacking. We used high temporal resolution hyperspectral imaging, liquid chromatography, pulse-amplitude fluorometry, oxygen microsensors, and confocal laser microscopy to study this response in a desiccated microbial mat from Exmouth Gulf, Australia. During the initial 15 min after rehydration chlorophyll *a* concentrations increased 2–5 fold and cyanobacterial photosynthesis was re-established. Although the mechanism behind this rapid increase of chlorophyll *a* remains unknown, we hypothesize that it involves resynthesis from a precursor stored in desiccated cyanobacteria. The subsequent phase (15 min–48 h) involved migration of the reactivated cyanobacteria toward the mat surface, which led, together with a gradual increase in chlorophyll *a*, to a further increase in photosynthesis. We conclude that the response involving an increase in chlorophyll *a* and recovery of photosynthetic activity within minutes after rehydration is common for cyanobacteria from desiccated mats of both terrestrial and marine origin. However, the response of upward migration and its triggering factor appear to be mat-specific and likely linked to other factors.

## Introduction

Microbial mats are dense benthic communities of diverse species of bacteria, archaea, and eukaryotic microalgae organized in a sediment matrix (Paerl et al., 2000; Franks and Stolz, 2009). In arid and mesic zones of the tropical, temperate and polar regions, photosynthetic microbial mats are the dominant biological feature (Whitton and Potts, 2012). In these systems, cyanobacteria are the dominant primary producers and display high rates of carbon and nitrogen fixation despite being active only during sporadic events of water availability (Lovelock et al., 2010).

Generally, microbial mats are laminated structures with microbial and physico-chemical zonation on millimeter or sub-millimeter scales (Franks and Stolz, 2009). Due to intense attenuation of light and mass transport limitations in the sediment matrix, microbial mats are characteristized by steep vertical gradients of oxygen, pH, and sulfide. These gradients change dramatically with diel periodicity due to variable input of light and these changes are generally thought to control the structure and distribution of the microbial communities in mats. Cyanobacteria have developed complex adaptations to cope with the dynamically changing micro-environments in microbial mats (Stal, 1995). For example, due to their critical dependence on light for photosynthesis, cyanobacteria migrate to optimize their position within the highly variable light field (Jørgensen and Des Marais, 1988; Al-Najjar et al., 2014), or to avoid the effects of self-shading, sedimentation, or exposure to strong ultraviolet radiation (Stal, 1995; Castenholz and Garcia-Pichel, 2002). The diel variations of light, oxygen, and sulfide are generally considered to be the key drivers for the motility of cyanobacteria in mats, although other factors such as salinity gradient (Kohls et al., 2010) and desiccation state (Pringault and Garcia-Pichel, 2004) have also been shown to play a role.

In arid cyanobacterial habitats such as desert crusts, the availability of water is the primary control of activity and migration (Garcia-Pichel and Pringault, 2001). A striking example of the adaptation of cyanobacteria to thrive in desert crusts is the rapid resumption of photosynthetic activity upon rehydration, which entails a ‘greening’ of the surface and can occur within minutes (Garcia-Pichel and Belnap, 1996). The green coloration of rehydrated desert crusts occurs due to the accumulation of photopigments at the surface, with some studies ascribing it to hydrotactic migration of cyanobacteria (Pringault and Garcia-Pichel, 2004) and other studies to the recovery of desiccated pigments (Harel, 2004; Abed et al., 2014).

While there are numerous studies on the rehydration response of terrestrial desert crusts and a few on sea-ice algae (Hawes et al., 1992), there are none that deal with desiccated marine microbial mats which face similarly parched conditions of extreme drought punctuated by sporadic availability of seawater. We hypothesized that a similar response occurs as in desert crusts, i.e., rapid reactivation of photosynthesis and upward migration to the mat surface. This idea is supported by the comparative study by Gray et al. (2007), which describes the similarities between desiccated micro-algae in marine and terrestrial systems and shows that phylogenetic relatedness is less important than environmental conditions for desiccation tolerance. We develop on this idea and attempt to integrate desiccated marine mats within the scope of desiccation studies of arid terrestrial systems. We studied the response of a desiccated cyanobacterial mat from Giralia Bay in Exmouth Gulf, Australia over the temporal scale of minutes-to-days after rehydration. We used minimally invasive techniques such as hyperspectral imaging fluorometry, and microsensors that allowed us to observe the rehydration response with a high temporal resolution.

## Materials and Methods

Hyperspectral imaging (HSI) was used as a non-invasive method of monitoring the chlorophyll *a* (chl *a*) content simultaneously at the surface and in the subsurface layers of the mat. The total chl *a* content was measured using high-performance liquid chromatography (HPLC), with a temporal resolution of minutes to days. Confocal laser scanning microscopy (CLSM) was employed to determine the fine-scale vertical distribution of the cyanobacterial filaments within the mat. To confirm that the revived cyanobacteria in the mats were photosynthetically active soon after rehydration and during migration, we measured the oxygen productivity within the mat using a microsensor following the light–dark shift technique (Revsbech et al., 1983). The photosynthetic potential was measured using pulsed-amplitude modulation fluorometry (PAM; Schreiber et al., 1994).

### Site Description and Sampling

The Exmouth Gulf in Australia, which is one of the largest unmodified arid zone estuaries in the world, has thick carpet-like desiccated cyanobacterial mats at the outer edge of the peritidal zone landward of the mangroves. The mats cover approximately 80 km^2^ in the eastern section of Exmouth Gulf, with an additional 20 km^2^ in the southern and western parts of the gulf (Lovelock et al., 2010). Analysis of the prokaryotic community using 16S rRNA sequencing indicated that the mats were dominated by cyanobacteria from the families *Chroococcaceae* and *Oscillatoriaceae* (Adame et al., 2012). Within the *Oscillatoriaceae* family, species from the highly cosmopolitan taxon *Microcoleus chthonoplastes* were dominant, with minor contributions of *Oscillatoria*. Furthermore, our microscopic investigations found abundant cyanobacterial filaments, whereas hardly any microalgae were found. A dark brown–green subsurface layer was observed which corresponded with the cyanobacterial community. The mats were sampled by excising the intact sediment surface with a pocket knife at various points along a 200 m transect within Giralia Bay (22.437°S, 114.34°E). The excised mats were placed on sand and allowed to dry naturally in full sunlight over 2 days. They were then wrapped in bubble foil and packaged for transport to the laboratory, where they were unpacked and placed in dry-air rooms maintained at 25°C for several months to establish complete desiccation prior to measurements. The desiccated mats were cut into approximately 1 cm × 1 cm pieces and inspected to verify that the subsurface brown layer of cyanobacteria were not damaged in the process. These mat pieces were used in rehydration experiments described below.

### Hyperspectral Imaging

We used HSI to study the response of the cyanobacteria to rehydration, by using protocols described previously by Chennu et al. (2013). Briefly, the system captures back-scattered light from the sediment and, using a spectral reference, converts the detected signal into reflectance spectra (wavelength range 400–900 nm, spectral resolution about 2 nm). These spectra were used to calculate a microphytobenthos index (MPBI) at each location in the spectral image. MPBI is a spectral index that is sensitive to the chl *a* concentration, as well as its vertical distribution, in the euphotic zone of the sediment (Chennu et al., 2013). A calibration between the “chl *a* signal” of MPBI and absolute chl *a* concentration was not attempted here due to the heterogeneity of the mat substrate with respect to the surficial crust, the laminar desiccated cyanobacteria, and the underlying grain sizes.

**FIGURE 1:**
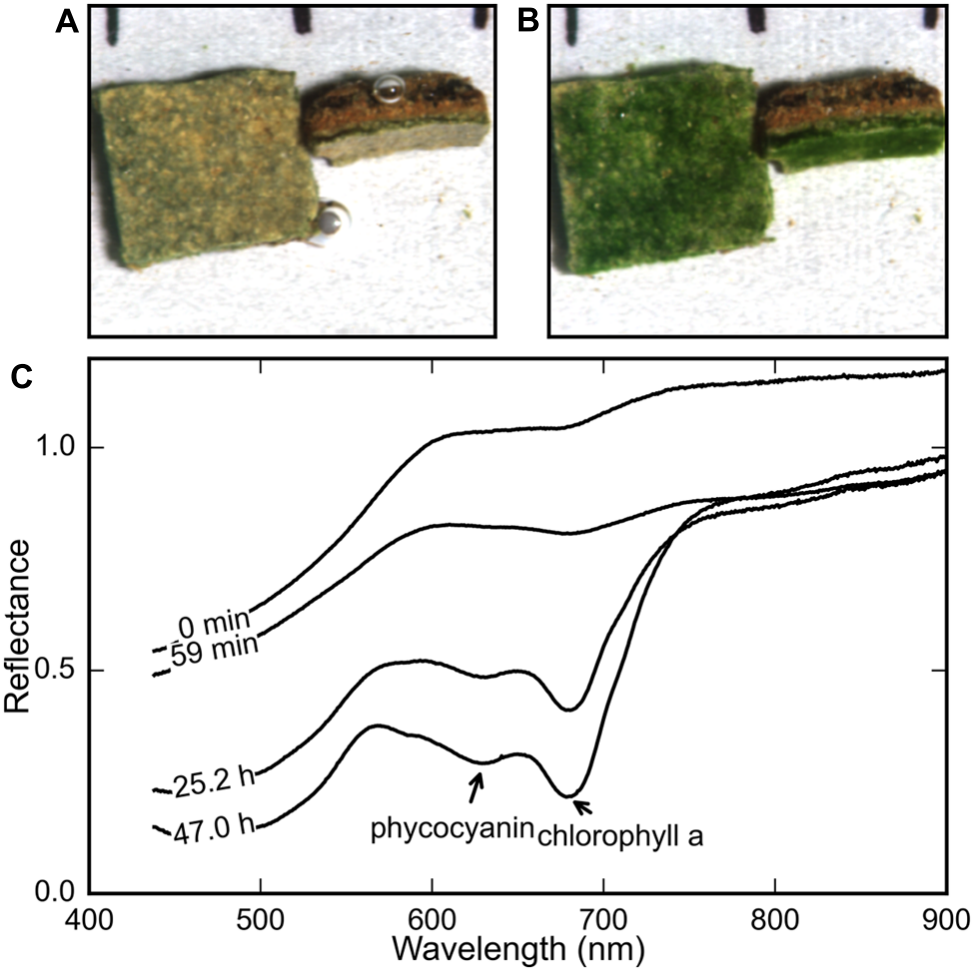
**Comparison of the true-color maps derived from hyperspectral images of a microbial mat piece **(A)** immediately after and **(B)** 2 days after rehydration (at *t* = 0 h) revealed the development of a bright green color in the surface and subsurface layers.** The average reflectance spectra of the rehydrated mat surface at various time-points **(C)** show the gradual development of spectral signatures of chl *a* (675 nm) and phycocyanin (630 nm).

Twelve mat pieces (prepared as described above) were fixed using modeler’s clay to a gray plastic board, such that the surfaces of nine mat pieces were parallel to the board. The other three pieces were fixed with their surfaces perpendicular to the board, exposing a cross-section of the mat’s deeper layers (**Figures 1A,B**). Together, these offered a simultaneous view of the surface and the subsurface layers of the mat. The reference board with the attached mat pieces was then placed in a large petri dish.

The HSI system was mounted on a linear motor about 50 cm above the Petri dish such that the area of the reference board together with the sample (5 cm × 5 cm) could be scanned in 1 min with a high spatial resolution (100 μm × 100 μm per pixel). The desiccated mats were scanned once. Then, to mimic rehydration of the mats due to tidal action in the field, seawater filtered through a 0.2 μm membrane was added to the Petri dish until a thin (5–10 mm) overlying layer of water formed above the mats. A minute after rehydration, the mats were scanned again, after which they were scanned periodically every 10 min for the first 1.5 h and thereafter every 2 h for a period of 2 days. Separate time-series of the rehydrated mat pieces were captured both under light and dark conditions. Illumination was provided by a halogen lamp attached to the imager, which was switched off between scans for the dark treatment.

The spectral images at each time point were used to generate true-color and chl *a* (MPBI) maps. The regions of the chl *a* map corresponding to the surface and subsurface layers of each mat were sectioned into separate regions-of-interest. For every time-point, the chl *a* signal values within each region were averaged and then compiled into a time-series for the subsurface and surface regions.

### High-Performance Liquid Chromatography

To determine the changes in the total chl *a* content of the mats during rehydration, we sampled the mat pieces at selected time-points and quantified the chl *a* content using HPLC. The entire mat piece was used as it was not possible to reliably separate the surface and subsurface layers. During the HSI monitoring, three mat pieces were collected at the following time-points: 0 min (desiccated), 15 min, 24 and 48 h for HPLC analysis. Additionally, to monitor the short-term changes in chl *a* content after rehydration, several small mat pieces (1 cm × 1 cm) were soaked in filtered seawater. Two of them were sampled each time in intervals of 2 min over a period of 20 min, in addition to 2 dry mat pieces to measure chl a content in the dry mat. Sampled mat pieces were crushed, added to 2 ml of 100% cold acetone, sonicated and placed for 24 h at -20°C to facilitate pigment extraction. Subsequently, the supernatant was filtrated through a 0.45 μm syringe filter (Acrodiscs CR 4 mm; Pall Gelman Laboratory, USA) and the filtrates were injected into a reverse-phase HPLC system consisting of a photodiode array detector (Waters 996) and a Waters 2695 separation module (Waters, Corp., USA). Pigments were separated using a 125 mm × 4.6 mm vertex column packed with Eurospher100 C18 particles of 5 mm in size (Knauer GmbH, Berlin, Germany). The chl *a* in the filtrates was quantified by comparing the retention time and spectrum of the eluents with respect to those of a chl *a* standard (DHI Water and Environment, Denmark), and normalized with respect to the volume (for the hyperspectral series samples) and to the weight (for the short-term series) of the mat pieces. The samples were kept on ice and under dim light during the measurement procedure.

### Confocal Laser Scanning Microscopy

To quantify changes in the vertical distribution of chl *a* in the mat after rehydration, a mat piece was measured using a CLSM system (Zeiss LSM 510), which consisted of an inverted microscope, a He–Ne (633 nm) photodiode laser, confocal scanner with photomultiplier tube and a computer to automate the measurements. The technique is based on measuring the fluorescent response centered around 660 nm of phycocyanin within the cyanobacterial filaments (Bittersmann and Vermaas, 1990; Poryvkina et al., 1994; Vermaas et al., 2008). With the initial focus of the optics leveled to the top surface of the mat and illumination at 633 nm provided by the He–Ne laser, a stack of Z-profile (up to a depth of 400 μm) images were collected over an area of over 1.2 mm × 1.2 mm with a fine voxel size (2.5 μm × 2.5 μm × 20 μm) within the desiccated mat. The light collected by the microscope was in two separate channels: one through a 650 nm long-pass filter to collect the fluorescent response of phycocyanin, and the other unfiltered to capture light reflected from the sediment matrix. Then, the mat was rehydrated with filtered seawater and the measurement scan repeated after 1 min. Thereafter, the mat remained untouched under the CLSM for a period of 24 h in the dark before being scanned again to a depth of 400 μm. The rehydration caused the mat to slightly expand (40–80 μm), which was taken into account in our analysis.

The extent of cell cover within the layers of the Z-stack was estimated by filtering the autofluorescence channel against a threshold (1.001) such that only pixels with filaments of cyanobacteria, and few detrital or noise pixels, were selected. The extent and intensity of the filtered pixels were considered proportional to the (pigmented) cyanobacterial biomass at those locations, irrespective of the depth within the mat. By summing the fluorescent intensity in each layer of the Z-stack, the density of pigmented biomass was determined (with a depth resolution of 20 μm) normalized and plotted against depth within the mat.

### Oxygen Microsensor Measurements

A desiccated microbial mat sample was placed in a small flow-through cell (11 cm × 4.5 cm × 5 cm) placed under a vertically incident collimated light beam from a tungsten-halogen lamp (KL 2500, Schott). The tip of a fast response Clark-type microelectrode (Revsbech, 1989) was positioned just above (200 μm) the surface of the dried mat using a microscope. Circulation of filtered seawater (3.5% salinity) was started within the flow-cell and measurements commenced after 5 min. This was to allow the hard crust of the mat to soften enough to allow microsensor tip penetration without damage.

Volumetric rates of gross photosynthesis (GP in mmol O_2_ m^-3^s^-1^) were measured using the light–dark shift technique (Revsbech et al., 1983), at incident irradiance of 320 μmol photon m^-2^s^-1^. GP measurements were conducted in vertical depth intervals of 100 μm, with three replicates at each depth, up to a depth of 600 μm. Vertical profiles of GP were obtained approximately every 15 min during the first hour of rehydration and every hour thereafter for a total of 12 h.

### Pulsed-Amplitude Modulation (PAM) Fluorometry

Photosynthetic potential within the mats were investigated using a PAM fluorometer (Walz GmbH, Germany) positioned 12 mm above the mat. Three different conditions of the mat were monitored: (1) dry mat under illumination, (2) a rehydrated mat under illumination, and (3) a rehydrated mat in the dark. The photosynthetic potential was measured using the pulse-saturation method (Schreiber et al., 1994, 1995; Kromkamp and Forster, 2003) with a pulse intensity of 2400 μmol photons m^-2^s^-1^ and duration of 0.8 s. The measured variable fluorescence response, which represents the yield of photosystem II, was sampled repeatedly in three replicate mat pieces over a period of 3 h after rehydration under the three treatment conditions.

## Results

In all our measurements, the color of the surface layer of the mat changed from brown to stark green over a period of 12–48 h after rehydration under both light and dark treatments. **Figures 1A,B** show the surface and side view of a mat at the start and end of a rehydration experiment. The stark green coloration at the end of the experiment (**Figure 1B**) provided a general confirmation that the desiccated mats responded to addition of filtered seawater, as described previously for terrestrial desert crusts (Pringault and Garcia-Pichel, 2004).

The reflectance spectra of the mat’s top and side surface were measured repeatedly using HSI during the course of a rehydration experiment. The average spectrum showed the gradual development of the spectral signatures of cyanobacterial photopigments chlorophyll *a* (chl *a*) and phycocyanin, with maximum *in vivo* absorption wavelengths at 678 and 630 nm, respectively (**Figure 1C**).

### Rapid Synthesis of Chlorophyll

**FIGURE 2:**
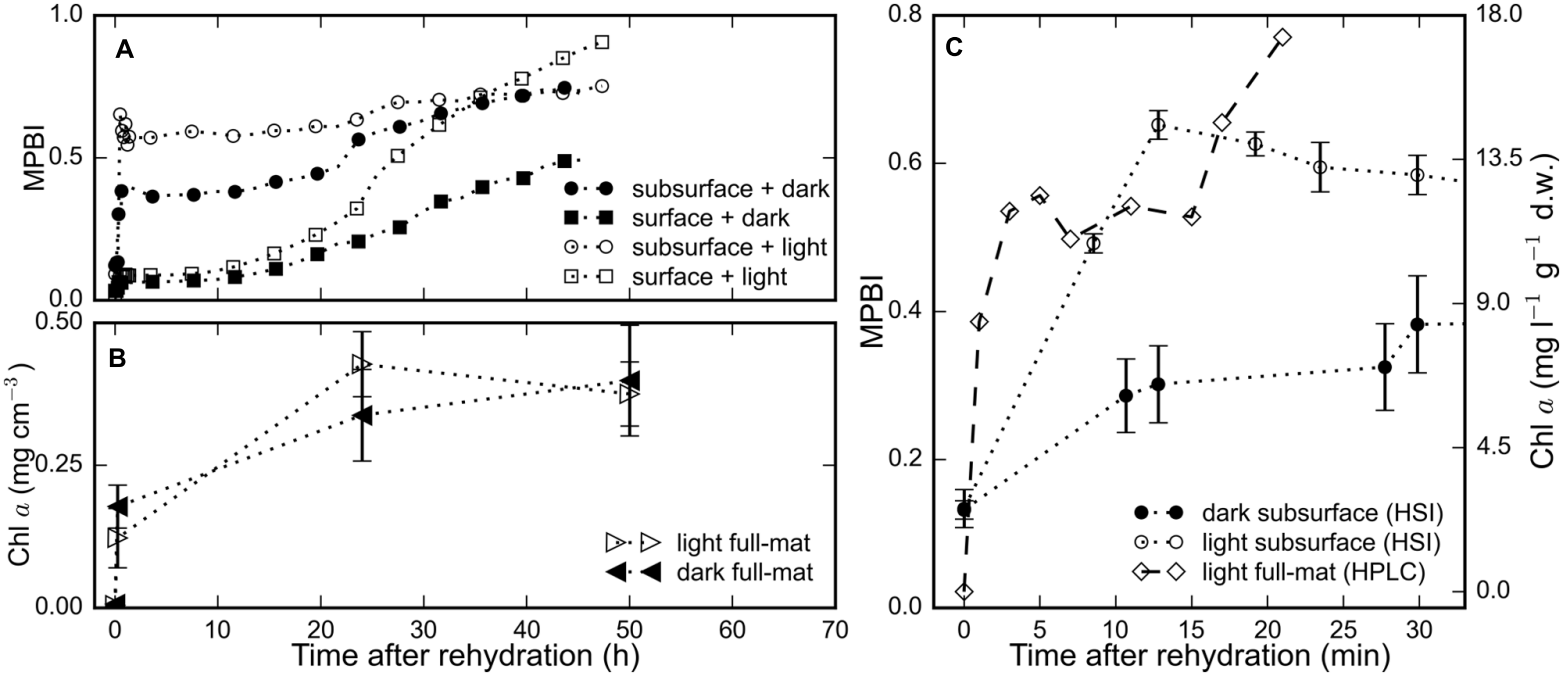
**The time-series of the chl *a* signal, determined as MPBI, averaged separately over the surface (*N* = 9) and subsurface (*N* = 3) layers of replicate mat pieces are shown in **(A)** for measurements in the dark (filled symbols) and in the light (open symbols).** **(B)** The total chl *a* content of a subset (*N* = 3) of the same mat pieces determined using HPLC. **(C)** Short-term changes in the total chl *a* content, as determined by HPLC, of (*N* = 2) different mats rehydrated (at *t* = 0 h) and maintained in the light is compared against the average subsurface chl *a* signal from the HSI time-series in **(A)** for both light (open circles) and dark (filled circles) treatments. Error bars represent standard errors.

The average chl *a* signal (MPBI) of the surface and subsurface regions of three replicate mats increased in response to rehydration under both light and dark treatments (**Figure 2A**). Within 15 min after hydration, we observed a steep increase in the chl *a* signal in the subsurface layers: with values higher by 4.9-fold in the light and by 2.3-fold in the dark (**Figures 2A–C**), whereas the values in the surface layer were only twofold higher for both light and dark treatments (**Figure 2A**). During the same period, the total chl *a* content of the full mat measured by HPLC increased by 1.7-fold (**Figures 2B,C**). Interestingly, the HPLC measurement could not detect any chl *a* in the desiccated state, but up to 11.9 mg l^-1^ g^-1^ d.w. was detected 2 min after rehydration. These results suggest that the recovery of photopigments in rehydrated microbial mats occurred within minutes.

### Rapid Reactivation of Photosynthesis

**FIGURE 3:**
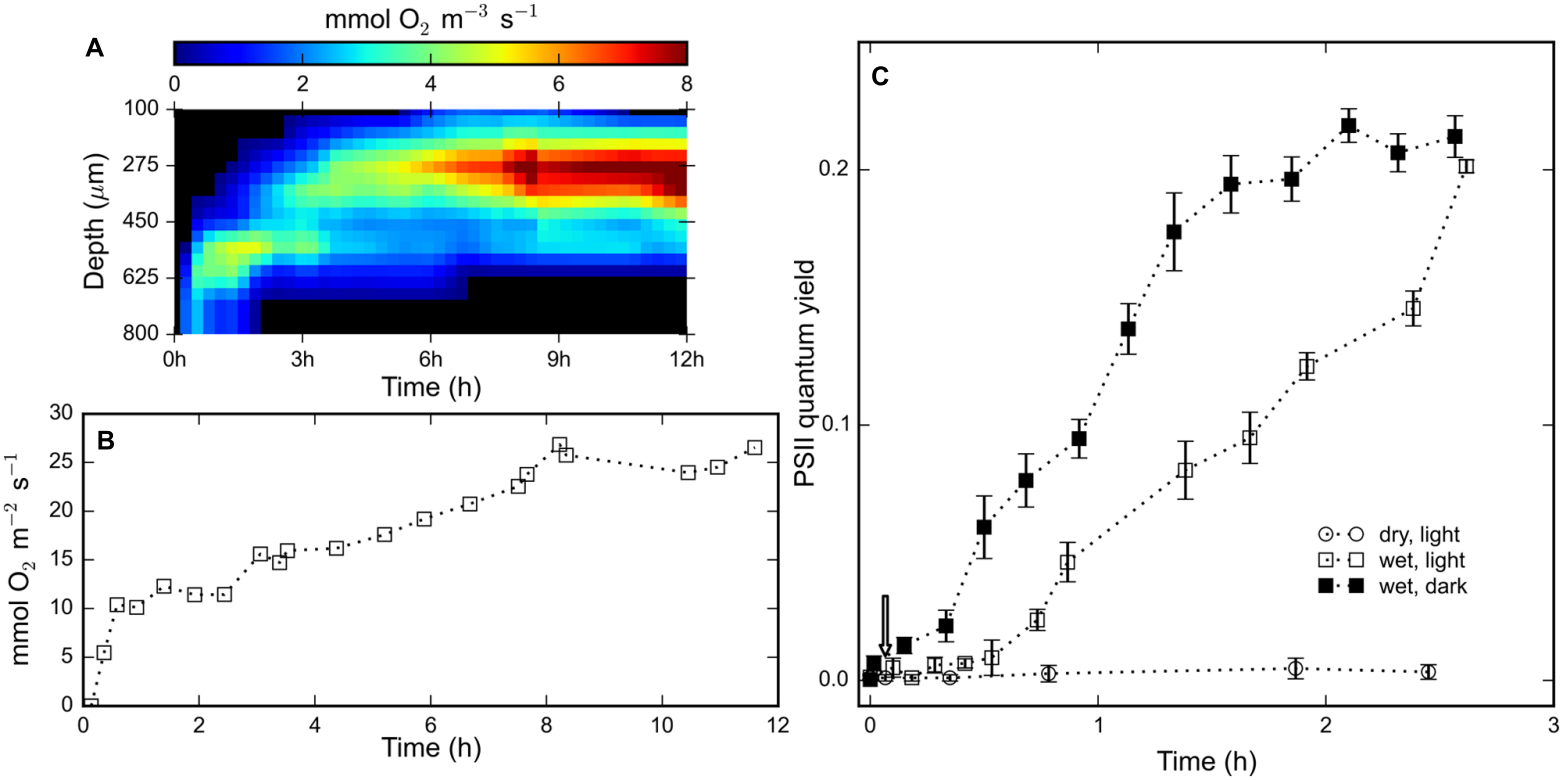
**(A)** Color-coded visualization of the time-series of depth profiles of the gross photosynthetic production within a rehydrated (at *t* = 0 h) mat piece as measured by the light–dark shift technique using an oxygen microsensor. **(B)** Depth-integrated oxygen production for the profiles shown in **(A)**. **(C)** Time-series of the average quantum yield of photosystem II measured using a PAM fluorometer of (*N* = 3) mat pieces maintained dry and illuminated (open circles), wet and illuminated (open squares) and wet and un-illuminated (filled squares). Error bars represent standard errors.

Time-series of depth profiles of the volumetric rate of gross photosynthesis were compiled into an array and visualized through a false-color image (**Figure 3A**). Photosynthetic oxygen production was detected about 13 min after rehydration at a depth interval of 600–800 μm. After 12 h, the width of this photosynthetic ‘band’ increased from about 200 μm to about 500 μm wide and the depth of the maximal rate of photosynthesis decreased from 700 to 400 μm (**Figure 3A**). The gradually widening and upward displacement indicated spreading and on average upward migration of the cyanobacterial population in the rehydrated mat. In the interval from 13 min to 12 h, the maximum volumetric rate of photosynthesis increased from about 2 mmol O_2_ m^-3^ s^-1^ to about 9.1 mmol O_2_ m^-3^ s^-1^ at about 8 h after rehydration (**Figure 3A**). The depth-integrated oxygen production increased steadily and reached a maximum of about 26.5 mmol O_2_ m^-2^ s^-1^ (**Figure 3B**). These results indicate that the rehydrated cyanobacteria engaged in photosynthesis within 15 min of rehydration, followed by gradual migration toward the surface.

The measurements from a PAM fluorometer showed no signs of pigment photoactivity in light-exposed desiccated mats (**Figure 3C**). The measured variable fluorescence (quantum yield of photosystem II) increased within 10 min after rehydration, under both light and dark conditions, and continued to rise for 2.5 h. The variable fluorescence of cyanobacteria in the mats in the dark was initially significantly higher than in the mats in the light, but after 2.5 h of increase mats from both light and dark treatments exhibited equal quantum yields (∼20%). The temporal evolution of the quantum yield is consistent with the observed oxygen production measured by microsensors (**Figure 3A**), which shows that cyanobacteria rapidly reactivate their photosystem II upon rehydration.

### Vertical Migration after Reactivation

**FIGURE 4:**
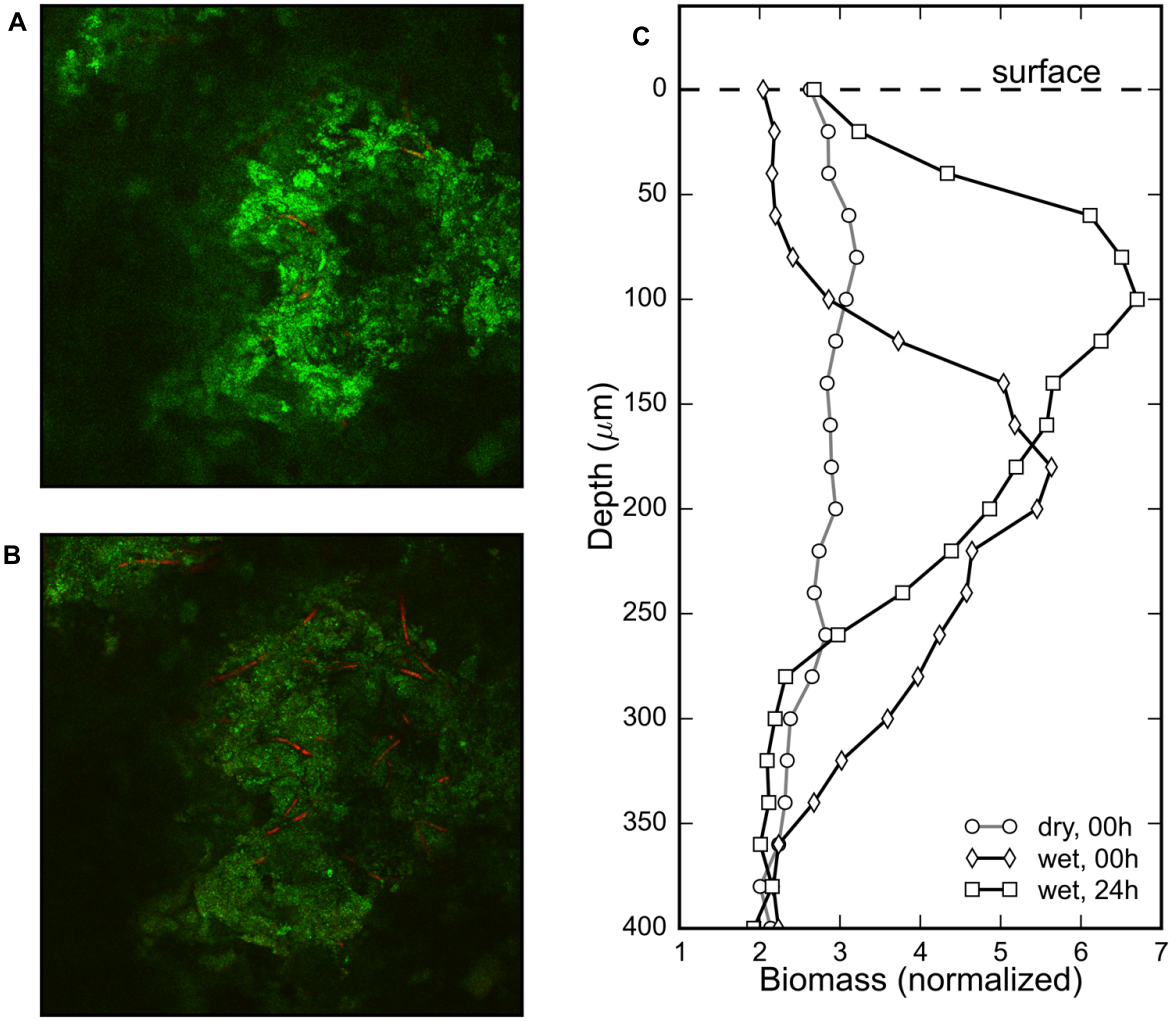
**Composite images of the sediment reflection (green) and phycocyanin autofluorescence (red) channels measured using a CLSM at the same depth (120 μm) of a cyanobacterial mat piece immediately after **(A)** and 24 h after **(B)** rehydration show increased density of cyanobacterial filaments in the latter image.** Depth profiles of the normalized (pigmented) biomass of cyanobacteria before, immediately after (<1 min) and 24 h after rehydration in **(C)** shows evidence of rapid appearance of phycocyanin and subsequent vertical migration of the reactivated cyanobacteria.

The gradual change in the surface color of the mat (**Figures 1A,B**) triggered by rehydration was considered to be an indication of the emergence of the revived cyanobacteria at the mat surface. To confirm the vertical displacement of the rehydrated cyanobacteria, the phycocyanin fluorescence signal in the Z-stack images from a CLSM was used to visualize the distribution of the cyanobacterial filaments within the mat. No significant phycocyanin signal could be detected in the desiccated mat. However, cyanobacterial filaments were visible a minute after rehydration (**Figure 4A**) and remained visible after 24 h in the dark (**Figure 4B**). Visual confirmation of the migration of the cyanobacterial filaments was possible by comparing these images from the two timepoints (**Figures 4A,B**).

The depth distribution of cyanobacterial filaments was assessed by comparing the profiles of the fluorescent biomass in a sample mat just before, immediately after and 24 h after rehydration (**Figure 4C**). The biomass profile of the dry mat was flat indicating that no fluorescent phycocyanin could be detected, despite the visible subsurface green layer in the mats (**Figure 1A**). The biomass profile immediately after (<1 min) rehydration showed a clear maximum centered around a depth of 200 μm, which after 24 h was centered around 100 μm (**Figure 4C**). These results show that cyanobacterial pigments buried within the mats begin to transition from photoinactive to photoactive within a minute upon rehydration, and subsequently migrate toward the mat surface over a period of 24 h.

## Discussion

The cyanobacterial mats from the arid estuary of Giralia Bay in Exmouth Gulf remain desiccated, on an average, for 280 days per year and receive water only sporadically due to unusually high tides or from rainfall, thus leaving only short periods when water is available for biological activity (Lovelock et al., 2010). Primary production by the resident cyanobacteria, upon which the entire mat ecosystem depends, is therefore limited to the short windows of opportunity (∼1 week) after hydration events. During inundation, they exhibit very efficient photosynthetic production (Al-Najjar et al., 2012), and fix up to 15% of the total carbon in the Exmouth Gulf (Lovelock et al., 2010). This suggests the action of specific adaptations in the cyanobacteria that facilitate an opportunistic lifestyle with respect to water availability, given that nutrients are abundant in the ecosystem.

The general observation among all the mats we studied was a brown–green subsurface layer and brown surface in the desiccated state, and the development of a stark green layer at the surface several hours after rehydration (**Figures 1A,B**). Our investigations attempted to document and understand the temporal sequence of the response of these desiccated cyanobacterial mats to rehydration. Our observations indicate that the rehydration response occurred in two phases: a rapid phase (1–15 min) consisting of resynthesis of photosynthetic pigment (**Figure 2**) and commencement of photosynthetic activity, followed by a gradual phase (15 min–48 h) that involved increase in photosynthetic production (**Figure 3**) and vertical migration (**Figures 3** and **4**) toward the mat surface.

The resynthesis of photopigments was rapid, and could be detected within a minute of rehydration in the subsurface cyanobacterial layer, as seen in the hyperspectral and HPLC time-series (**Figure 2C**) for chl *a* as well as in the CLSM data for phycocyanin (**Figure 4**). The detection of variable fluorescence (**Figure 3C**) and phycocyanin fluorescence (**Figure 4C**) within minutes after rehydration, while none was detected in the desiccated mats, indicates the rapid reactivation of the photosynthetic apparatus of the rehydrated cyanobacteria. It is worth noting that no chl *a* could be detected from the HPLC measurements in the desiccated mat, whereas ∼9 mg chl *a* l^-1^ g^-1^ d.w. was measured just 2 min after rehydration (**Figure 2C**). This is unlikely to be due to a methodological problem of extracting pigment from the dry mat since most of the pigment is usually extracted after 24 h in acetone, and is further supported by the biomass profiles measured by CLSM immediately before and after rehydration (**Figure 4C**). Therefore, we exclude that chl *a* was formed from *de novo* production since the synthesis time of chl *a* or the regeneration time of *Microcoleus* is far longer (Beale and Appleman, 1971; Tiwari et al., 2001). This was confirmed by Abed et al. (2014) in terrestrial desert crusts. Together, these suggest that a rapid resynthesis of photosynthetic pigments occur upon rehydration in both desiccated marine microbial mats and desert crusts.

Photosynthetic activity was first detected 13 min after rehydration and increased over several hours, as seen in the oxygen production and PAM fluorescence measurements (**Figure 3**). These rapid responses occurred both when the mat was placed under light or in the dark (**Figures 2–4**), although with different rates suggesting some light-dependance of the process. In the case of PAM fluorometry, the higher quantum yields of mats placed in the dark is expected as the plastoquinone pool between the two photosystems is less occupied by electrons than for the cells under light.

The gradual response to rehydration consisted of an increase in photosynthetic capacity and production of the cyanobacteria over a period of 12 h (**Figure 3**), concomitant with vertical migration toward the mat surface. The vertical migration of the cyanobacterial filaments, visually confirmed from CLSM images and threshold-filtered biomass profiles (**Figure 4**), led to the stark green mat surface after 48 h (**Figure 1**). The combined effect of migrating along the light gradient and restoration of photosynthetic activity is that the oxygen productivity of the cyanobacterial mat gradually increased by about 500% over a period of 12 h (**Figures 3A,B**). Vertical migration is a common motility adaptation of cyanobacteria to optimize their position within the light field (Garcia-Pichel et al., 1994; Bebout and Garcia-Pichel, 1995). For cyanobacteria, motility is known to be driven by a variety of factors such as salinity (Kohls et al., 2010), desiccation state (Pringault and Garcia-Pichel, 2004) or photo-avoidance (Castenholz and Garcia-Pichel, 2012). In our case, we did not identify the exact drivers for the vertical migration and we speculate that it is likely driven by salinity gradients set up during evaporation and rehydration.

A number of reports exist that deal with the reactivation of photosynthesis in desert soil crusts, while relatively few exist about desiccated marine microbial mats. As Gray et al. (2007) note, these ecosystems are comparable despite facing different meteorological conditions because they are both fundamentally reliant on the adaptations of the desiccated cyanobacteria/algae that allow rapid reactivation of photosynthesis during sporadic water availability. We note that photosynthetic production was measured in various desert crusts within 10–15 min of rehydration by Garcia-Pichel and Belnap (1996), Péli et al. (2011), Abed et al. (2014), and Lan et al. (2014), which is directly comparable to our observations with desiccated marine microbial mats. However, we also note several dissimilarities between the motility adaptations in desert crust studies and our own. Garcia-Pichel and Pringault (2001) described a sensitive hydrotactic response in desert crust cyanobacteria, whereas Abed et al. (2014) observed no vertical migration upon rehydration. This may be due to a difference in the specific adaptation of the samples, for example to light intensities (Gray et al., 2007), or possibly due to problems with interpretation of migration. Deriving migration from oxygen profiles could be confounded by temporal changes in activity (Garcia-Pichel and Belnap, 1996), whereas spectrometric interpretation of migration may be confounded by heterogeneous vertical distribution of the pigmented biomass (Chennu et al., 2013). The latter issue is possibly relevant for our HSI time-series (**Figure 2**). However, we confirmed the physical displacement of the cyanobacterial cells in our samples with CLSM imaging, and found this process to occur simultaneously but at a much slower (hours-to-days) rate than photosynthetic reactivation (minutes). Based on the literature and our observations, we recommend that future studies of cyanobacterial reactivation measure photosynthetic activity and migration separately, with adequate temporal and spatial resolution to capture both processes.

In both terrestrial and marine ecosystems, desiccation imposes severe structural, biochemical and physical stress on cells. The ability of cyanobacteria and algae to overcome extreme desiccation is reliant on the physiological adaptation to the environment irrespective of their phylogenetic relatedness (Gray et al., 2007). The rapidity of the reactivation of photosynthesis is remarkable, but remains poorly understood. Several factors have been tested such as temperature during desiccation or water content of the cells (Péli et al., 2011; Lan et al., 2014). It is clear that the time required to reactivate photosynthesis (5–15 min) is not enough for *de novo* synthesis of required proteins or photopigments (Harel, 2004; Abed et al., 2014). It is likely that the desiccation induces the photopigments in the cells to be broken down and recycled (Vavilin and Vermaas, 2007), which could then be stored in some stable form for reactivation (Billi and Potts, 2002). We hypothesize that the cyanobacteria store the chlorophyll in a precursor form, most likely protoporphyrin IX, which involves removing the magnesium ion from the ring structure and upon subsequent rehydration this ion is quickly re-inserted to form chlorophyll (Jensen et al., 1999). This is supported by field measurements of Lovelock et al. (2010), which found twofold higher magnesium concentration in the cyanobacterial mat sediments compared to the ambient sediments. The hypothesis and the mechanism of the adaptation remain to be fully explained.

Overall, recent investigations by Rajeev et al. (2013) reveal the variety of mechanisms that desiccated cyanobacteria possibly engage upon facing desiccation: glycogen breakdown as an alternative energy source, increased reactive-oxygen-species scavengers as a protection against light damage, preparation of metabolic intermediates, storage of exopolysaccharides, maintenance of mRNA, etc. Furthermore, it has been shown that cyanobacteria reuse the organic carbon stored in extracellular polymeric substances (Stuart et al., 2015), which would further aid their survival through a long dormancy. These findings motivate further proteomic studies of the remarkable adaptations of cyanobacteria to survive extreme desiccation in both terrestrial and marine habitats.

In summary, using a variety of techniques we monitored, with high spatial and temporal resolution, the sequence of events taking place in response to rehydration of desiccated marine cyanobacterial mats. The rehydration response starts with rapid chl *a* resynthesis that occurred within 1–2 min after rehydration. This was followed by reactivation of photosynthesis occurring after 13 min, followed by subsequent vertical migration over several hours. This response sequence was observed in both light and dark conditions, and is directly comparable to previous studies of cyanobacteria in terrestrial desert crusts. The rapidity of the reactivation of photosynthesis leads us to hypothesize the presence of an adaptation in cyanobacteria that allows them to sequester their chl *a* in a precursor state during desiccation. The ability to sequester and rapidly reactivate the photosynthetic apparatus are likely crucial adaptations that enable cyanobacteria to thrive in arid environments with sporadic water availability. This remarkable ability allows them to reactivate primary production from a desiccated state, and thereby provide sustenance to the co-dependent microbial communities. With their finely tuned adaptation of photosynthetic reactivation and vertical migration, cyanobacteria opportunistically exploit the rare availability of water to revive the function of desiccated ecosystems in both terrestrial and marine habitats.

## Author Contributions

The study was conceived by MAl-N and AC, with design input from LP and DdB. Experimental measurements were conducted by AC, MAl-N, and AG. Data analysis and interpretation was done by all authors. Preparing the manuscript and reviewing was done by all authors.

## Conflict of Interest Statement

The authors declare that the research was conducted in the absence of any commercial or financial relationships that could be construed as a potential conflict of interest.
